# Clinical identification of bacteria in human chronic wound infections: culturing vs. 16S ribosomal DNA sequencing

**DOI:** 10.1186/1471-2334-12-321

**Published:** 2012-11-24

**Authors:** Daniel D Rhoads, Stephen B Cox, Eric J Rees, Yan Sun, Randall D Wolcott

**Affiliations:** 1 Department of Pathology, University of Pittsburgh Medical Center (UPMC), Pittsburgh, PA, USA; 2 Research and Testing Laboratory (RTL), Lubbock, TX, USA; 3 Southwest Regional Wound Care Center (WCC), Lubbock, TX, USA; 4 PathoGenius Laboratories (PGL), Lubbock, TX, USA

**Keywords:** Pressure ulcer, Diabetic foot, Molecular diagnostic techniques, Bacteria, Microbiology, 16S

## Abstract

**Background:**

Chronic wounds affect millions of people and cost billions of dollars in the United States each year. These wounds harbor polymicrobial biofilm communities, which can be difficult to elucidate using culturing methods. Clinical molecular microbiological methods are increasingly being employed to investigate the microbiota of chronic infections, including wounds, as part of standard patient care. However, molecular testing is more sensitive than culturing, which results in markedly different results being reported to clinicians. This study compares the results of aerobic culturing and molecular testing (culture-free 16S ribosomal DNA sequencing), and it examines the relative abundance score that is generated by the molecular test and the usefulness of the relative abundance score in predicting the likelihood that the same organism would be detected by culture.

**Methods:**

Parallel samples from 51 chronic wounds were studied using aerobic culturing and 16S DNA sequencing for the identification of bacteria.

**Results:**

One hundred forty-five (145) unique genera were identified using molecular methods, and 68 of these genera were aerotolerant. Fourteen (14) unique genera were identified using aerobic culture methods. One-third (31/92) of the cultures were determined to be < 1% of the relative abundance of the wound microbiota using molecular testing. At the genus level, molecular testing identified 85% (78/92) of the bacteria that were identified by culture. Conversely, culturing detected 15.7% (78/497) of the aerotolerant bacteria and detected 54.9% of the collective aerotolerant relative abundance of the samples. Aerotolerant bacterial genera (and individual species including *Staphylococcus aureus*, *Pseudomonas aeruginosa*, and *Enterococcus faecalis*) with higher relative abundance scores were more likely to be detected by culture as demonstrated with regression modeling.

**Conclusion:**

Discordance between molecular and culture testing is often observed. However, culture-free 16S ribosomal DNA sequencing and its relative abundance score can provide clinicians with insight into which bacteria are most abundant in a sample and which are most likely to be detected by culture.

## Background

Chronic wounds impact the health of over 8 million people in the United States each year, and the direct healthcare associated cost of these wounds is over 25 billion of dollars each year [1]. These chronic wounds include venous stasis ulcers, diabetic foot ulcers, decubitus ulcers, and non-healing surgical wounds. Medicine has worked to address the many barriers to healing including repetitive trauma, poor nutrition, impaired host defense, and infection. The presence of bacterial biofilms in chronic wounds has been established [2], and wound care clinicians attempt to manage these bacteria in chronic wounds [3]. In this study, we examine the bacterial components of the wound bioburden using two clinical laboratory tests: traditional aerobic culturing and recently developed culture-free sequencing of bacterial 16S DNA.

Previous studies have compared culturing to molecular methods [2,4-10], and we have performed studies to compare culturing to the molecular testing method we use in this study [2,9]. These studies agree that molecular microbial tests are more sensitive than culture testing and hold promise for improving patient care. The current study adds to this body of work. The molecular test used in this study produces a relative abundance score, and we describe the relevance of this score and the correlation it has to culture results.

Providing more accurate information to the clinician via more sensitive testing is typically the goal of the clinical microbiology laboratory. More sensitive microbial testing, such as culture-free sequencing of bacterial DNA that is used in this study, can identify numerous microbial taxa in a single clinical sample. Unfortunately, the clinician can have difficulty discerning which bacteria could be most clinically relevant. Being able to correlate the results of this newer testing method with a familiar testing method (culturing) would be helpful for the clinician. Culture-free sequencing of bacterial DNA reveals qualitatively which bacteria are present, but it also reports a relative abundance score for each organism. Correlating this relative abundance score with the likelihood that the same bacterium would be detected by culture has not been performed until now. Correlating the results of molecular microbial diagnostic tests with the results from culturing will give clinicians insight into understanding these new tests and their clinical relevance. Improving clinicians’ ability to interpret test results will enable them to better customize their patient care and overcome each individual patient’s barriers to healing.

## Methods

In this study, the chronic wounds from 51 subjects were sampled for study in accordance with Western Institutional Review Board protocol #20062347. All subjects signed informed consent documents. All samples were obtained at the Southwest Regional Wound Care Center (Lubbock, Texas) in 2009. Types of wounds included chronic wounds such as venous stasis ulcers, diabetic foot ulcers, pressure ulcers, and non-healing surgical wounds. Each sample of devitalized tissue was obtained using sharp debridement as part of standard of care. Each chronic wound site was sampled in duplicate for parallel testing. One portion was analyzed by culture in an academic, hospital-based microbiology laboratory while the other portion was analyzed by molecular methods at PathoGenius laboratory (Lubbock, Texas). Both labs are accredited by the College of American Pathologists.

### Criteria for inclusion and exclusion

Fifty-one (51) consecutive subjects that presented to the Southwest Regional Wound Care Center with full thickness wounds (e.g. venous stasis ulcers, diabetic foot ulcers, decubitus ulcers, and non-healing surgical wounds) were included in the study if they had a chronic wound as defined as a wound that fails to progress through normal wound healing trajectory, if sharp debridement was required as part of their standard of care, and if enough debridement material was obtained in order to perform both culture and molecular testing.

### Sample collection and processing

Cultures were obtained as per laboratory protocol. Samples from the host-bioburden interface were obtained at the Southwest Regional Wound Care Center and transported to the hospital microbiology laboratory for aerobic culture and identification. Some of the isolated bacteria were then analyzed by the Research & Testing Laboratory (Lubbock, Texas, USA) to verify the isolates’ identities using molecular testing. A second, parallel sample was obtained and placed in a 2 mL microcentrifuge tube and tested by Pathogenius (Lubbock, Texas, USA).

### 16S Ribosomal DNA (rDNA) processing & analysis

The methods have been thoroughly described previously [9]. Briefly, DNA was extracted from the samples, and the 16S portion of ribosomal DNA was amplified. The 16S amplicons were then pyrosequenced using Roche’s (formerly 454’s) FLX Titanium technology, and sequences were queried against a taxonomic database of high quality sequences derived from NCBI using BLASTN+. Sequences with identity scores to well characterized 16S sequences greater than 97% identity (<3% divergence) were resolved at the species level, between 95% and 97% at the genus level.

### Determining the contribution of each bacterial taxon to the wound microbiota

The total number of sequence matches for an organism in each sample was considered 100% of the relative abundance for that sample. Most of the analysis was performed at the genus level, but some analysis was also performed at the species level (*Staphylococcus aureus*, *Pseudomonas aeruginosa*, *Enterococcus faecalis*). Each detected taxon comprised a percentage of the total relative abundance in the sample. For some of the analyses, these percentages were divided into three categories based upon the frequency of taxon detection. Taxa with a relative abundance of more than 10% were considered to be “Dominant” contributors to the sampled microbiota. Taxa with relative abundance of 1-10% were considered to be “Major” contributors to the sampled bacterial population, and taxa with relative abundance of less than 1% were considered to be “Minor” components of the bacterial population in the wound. This arbitrary scoring system was employed to the genera within the dataset.

### Cross-checking of culture and molecular results

Research & Testing Laboratory (Lubbock, Texas) was used to cross-check the culture isolates using molecular testing. The clinical microbiology laboratory provided cultured isolates to the research laboratory for verification of identification. The research laboratory extracted 16S DNA and sequenced a portion of the gene to determine each isolate’s species. The bacterial species determined by molecular testing in the research laboratory was then compared to the clinical laboratory’s identification of the isolate.

### Statistical methods

The combined microbiome of all samples was illustrated using a rank-abundance plot. This plots each genus’ relative abundance (assessed via molecular methods) as a function of its rank within the entire community, and graphically illustrates the overall diversity of the wound microbiome. The relationship between the likelihood of a bacterium being cultured and its relative abundance was graphically illustrated using a mosaic plot and was statistically evaluated using logistic regression. Specifically, we used a generalized linear model that assumed a binomial error distribution and a logit link function. Significance of the relationship was assessed using a likelihood ratio test, and results are reported as an odds ratio with a 95% confidence interval. The odds ratio is a measure of the increase in the odds of being detected via culture based methods given an increase in relative abundance. Statistical analyses were conducted in R [9].

## Results & discussion

Like previous studies, this study demonstrates that culture testing and molecular testing of wound bacteria produce differing and often conflicting results because molecular methods are more sensitive than culture methods [7-10]. The current study demonstrates that it is important to consider the relative abundance of the bacteria that are detected by the molecular test used in this study and not only the qualitative results of the test. The relative abundance of the bacterial species within the sample helps to predict the likelihood that the species will be detected by culture. That is, bacteria with greater relative abundance within a wound sample are more likely to be cultured than other bacteria with less relative abundance. These findings are different from what has been previously reported in a study using a different molecular method of analysis (fluorescent *in situ* hybridization), which reported, “the absence of a correlation between the bacterial species *Staphylococcus aureus* and *Pseudomonas aeruginosa* detected in wound by culture and what is in fact present” [5].

In the current study, 51 samples were cultured, and 93 isolates were identified. One sample yielded two species from the same genus, so 92 genus-level taxa were identified in the 51 samples. An average of 1.8 ± 0.9 bacterial genera were identified in each sample (Table 1), representing 14 unique genera in total. *Staphylococcus* and *Enterococcus* comprised the majority of the detected genera; they were detected 28 and 21 times, respectively. When considering only the 92 detected genera, 16S DNA sequencing identified the same genus in the sample 78 times. Of those 78 times, the bacterial population was determined to be a dominant population (>10% of the wound microbiota relative abundance) 43 times, a major population (1-10% of the wound microbiota relative abundance) 18 times, and a minor population (<1% of the wound microbiota relative abundance) 17 times. Fourteen (14) times, the bacterial genus identified by culture was not detected by molecular testing. It is possible that some of the 14 bacterial genera that were identified by culture but not identified by 16S testing may have been misidentified by one of the testing methods (Table 2). Genetic testing is the gold standard for species identification, so if one of the tests misidentified an organism, it is most likely that culturing misidentified the organism. Another possibility is that deeper 16S DNA sequencing would have revealed the genera that were identified by culture.

**Table 1:** Comparison of the number of bacterial taxa detected using molecular testing and culturing testing

	**Mean**	**STD**	**Min**	**Max**
**16S Testing for All Bacterial Genera**				
All Genera	14.8	7.5	3	38
Dominant Genera	2.2	1.1	1	5
Major Genera	3.7	3.7	0	16
Minor Genera	8.9	5.7	0	26
**16S Testing for Aerotolerant Bacterial Genera**				
All Genera	9.8	5.3	2	29
Dominant Genera	1.8	0.9	0	4
Major Genera	2.4	2.3	0	10
Minor Genera	5.6	4.4	0	19
**Bacterial Genera Detected by Aerobic Culture**				
All Genera	1.8	0.9	1	5

The number of bacterial genera identified using molecular testing and the number of bacterial genera cultured using aerobic testing of the 51 samples are listed. The mean number of genera identified using molecular identification or isolates identified using culture identification are listed. Molecular testing results are listed for both 1) all bacterial genera and for 2) only aerotolerant bacterial genera. The standard deviation (STD), minimum number of genera (Min), and the maximum number of genera (Max) are also listed. These same data were determined for subsets of genera determined to be present at >10% of the relative abundance in the sample (Dominant), 1-10% of the relative abundance in the sample (Major), and less than 1% of the relative abundance in the sample (Minor).

**Table 2:** Discrepant results between bacterial isolate identification by phenotype and isolate identification by genotype

**Phenotypic Identification**	**Genetic Identification**
*Enterobacter cloacae*	*Escherichia coli*
*Pseudomonas aeruginosa*	*Salmonella enterica*
*Staphylococcus* spp.	*Enterococcus faecalis*
Coagulase Negative Staphylococci	*Enterococcus faecalis*
*Aeromonas hydrophila*	*Aeromonas punctata*
*Citrobacter freundii*	*Citrobacter murliniae*
*Enterococcus raffinosus*	*Enterococcus avium*
*Enterococcus raffinosus*	*Enterococcus avium*
*Staphylococcus intermedius*	*Staphylococcus pseudintermedius*

Of the 46 cultured bacterial isolates that were cross-checked using DNA testing to verify identification, discrepancies in the identification of the isolate were present 9 times. The 9 discrepant isolates are listed above. The biochemically-determined taxonomic classification determined by culture phenotype is in the left column, and the molecularly-determined species classification by DNA 16S analysis is in the right column.

**Figure 1:**
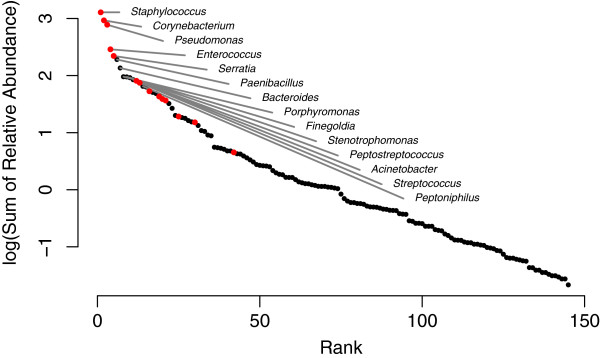
**Frequently identified bacteria genera.** Rank abundance distribution of the 145 bacterial genera detected by 16S DNA sequencing methods. Here, abundance is based on the sum of relative abundances from all samples. Red dots represent genera that were cultured aerobically. Only the 14 most abundant genera are labeled.

**Figure 2:**
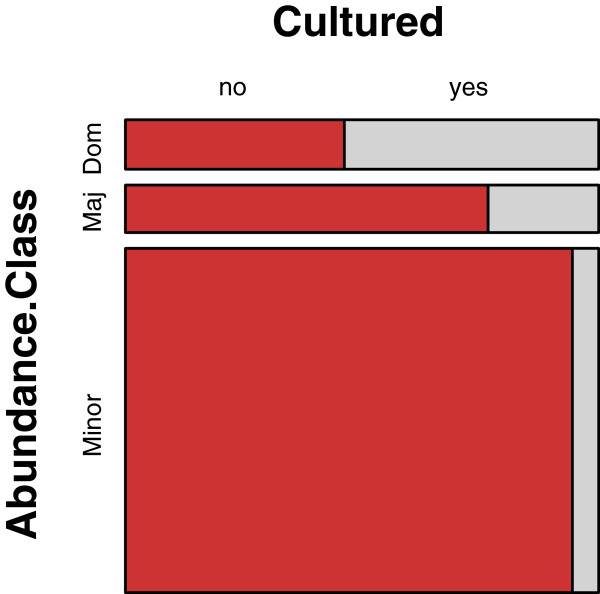
**Relative abundance of wound bacteria influences their likelihood of culture detection.** Mosaic plot of the number of times a detected bacterial genus (as determined by molecular methods) was cultured (as shown in gray). Only aerobic bacteria were considered, and bacterial genera were divided into dominant (>10%), major (1-10%), or minor (<1%) components of each sample based upon their relative abundance within each sample. Areas of each component of the plot are proportional to the observed number of occurrences. Dominant genera were cultured 47% (43/91) of the time. Major genera were cultured 15% (18/120) of the time. Minor genera were cultured 5.9% (17/286) of the time.

16S sequencing methods resulted in an average of 2411 sequences per sample, with the average read length for all samples being 466 bases. From these sequences, 753 bacterial taxa (genera) were detected (average number per sample, 14.8 ± 7.5), and these 753 positive results were found within 145 unique genera (Figure 1, Table 1, and Additional file 1). Forty-seven percent (47%; 68/145) of the unique genera were aerotolerant and potentially culturable using aerobic culture methods. Sixty-six percent (66% ; 497/753) of the positive results were attributed to the 68 aerotolerant genera (average per sample 9.8 ± 5.3), and these were the bacteria considered in most of the analyses. Sequence analysis identified 18% (91/497) of the bacteria as dominant components of the population, 24% (120/497) as major components to the population, and 58% (286/497) as minor components of the population. Aerobic culturing detected 15.7% (78/497) of the potentially culturable organisms. Cultures identified dominant genera 47% (43/91) of the time, major genera 15% (18/120) of the time, and minor genera 5.9% (17/286) of the time (Figure 2).

It can be argued that the 58% (286/497) of results attributed to bacteria that occurred with less than 1% relative abundance in their samples were environmental contaminants within the wounds. If this is the case, it is important to note that 18% (17/92) of the bacteria that were isolated by culture were in this group, so culturing also detects bacteria that may be environmental contaminants. Another 15% (14/92) of the cultured bacteria were not detected at all using molecular methods, which could be because of very low levels of the bacteria within the wound. In total, one-third (31/92) of the genera detected by culture were either not detected using 16S sequencing or detected to be present at less than 1% of the relative abundance within the sample. The clinical usefulness of attempting to manage bacteria present at such low abundance within a sample is questionable. The molecular testing results suggest that culture testing may be insufficiently sensitive in some cases or overly sensitive in other cases. Therefore, careful clinical interpretation of culture results is necessary [11,12].

**Figure 3:**
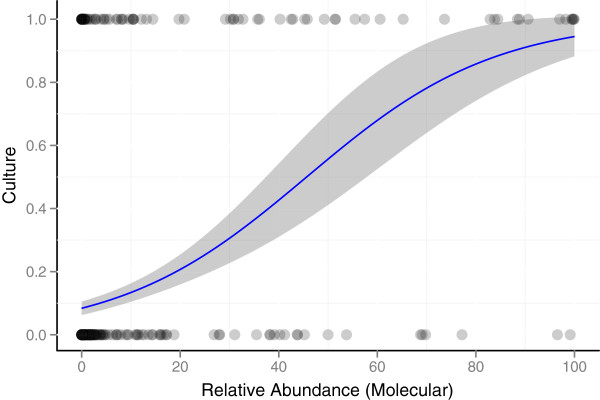
**Increased relative abundance of a bacterial genus increases the likelihood that the genus will be cultured.** Each dot represents the detection of a bacterial genus by 16S DNA sequencing. Genera that were detected by culture (response = 1) are represented by dots at the top of the figure, and genera that were not cultured (response = 0) are represented as dots at the bottom of the figure. The proportion of occurrences that were detected by culture was modeled as a function of the observed relative abundance (as determined using molecular testing) using logistic regression (indicated by the blue line). The results demonstrate that an increased relative abundance of a bacterial genus subsequently increases the likelihood that the genus will be cultured. The shaded region indicates the 95% confidence interval of the predicted proportion.

Some bacteria were more commonly detected by culture than others (Table 3). In some instances, this variation in detection can be attributed to the relative abundance of the bacteria in the original sample (Figures 2, 3, &4), but detection also depends upon the type of bacteria. For example, culturing identified *Staphylococcus* in 61% (28/46) of the samples that were positive by molecular testing (average relative abundance of 28% per sample), but *Corynebacterium* was only identified by culture in 16% (6/38) of the samples that were positive by molecular testing (average relative abundance of 24% per sample). Both the organism and its relative abundance affect the ability to detect the organism by culture.

**Table 3:** Comparison of the frequently identified bacterial genera detected by molecular and culture testing

**16S DNA Sequencing**		**Culture**	
**Samples**	**Genus**	**Samples**	**Genus**
46	Staphylococcus*	28	Staphylococcus*
38	Corynebacterium*	21	Enterococcus*
32	Pseudomonas*	8	Pseudomonas*
31	*Clostridium*^*†*^	8	Serratia*
28	Bacillus*	7	*Proteus*
27	Enterococcus*	6	Corynebacterium*
26	*Paenibacillus*	4	Enterobacter*
22	Serratia*	2	Citrobacter*
22	*Propionibacterium*^*†*^	2	Escherichia*
20	Escherichia*	2	Streptococcus*
20	Streptococcus*	1	Acinetobacter*
19	*Brevibacterium*	1	*Aeromonas*
17	Citrobacter*	1	Bacillus*
17	Klebsiella*	1	Klebsiella*
17	*Lactobacillus*		
17	*Finegoldia*^*†*^		
15	*Sarcina*^*†*^		
13	*Anaerococcus*^*†*^		
11	*Bacteroides*^*†*^		
11	*Peptoniphilus*^*†*^		
11	*Prevotella*^*†*^		
10	Enterobacter*		
10	*Salmonella*		
9	*Providencia*		
9	*Peptostreptococcus*^*†*^		
8	Acinetobacter*		
8	*Nocardiopsis*		

Of the 145 genera identified by 16S sequencing in the 51 samples in this study, the 27 most frequently identified genera are included in the table. Nine (9) of these 27 genera are obligate anaerobes, and culture did not attempt to detect these organisms. All 14 of the bacterial genera identified by culture are included in the table. The two bacteria detected by culture and not listed in the “16S DNA Sequencing” column of the table are *Proteus* and *Aeromonas*, which were detected using 16S sequencing 6 and 1 times, respectively. The frequency of identification of each genus is noted under the “Samples” heading.

* Genus present in both columns.

^*†*^ Obligate anaerobic genus.

**Figure 4:**
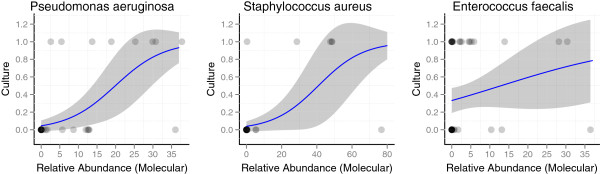
**Increased relative abundance of a bacterial species increases the likelihood that the species will be cultured.** Each dot represents the detection of the species by 16S deep sequencing. Species that were detected by culture (response = 1) are represented by dots at the top of the figure, and species that were not cultured (response = 0) are represented as dots at the bottom of the figure. The proportion of occurrences that were detected by culture was modeled as a function of the observed relative abundance (as determined using molecular testing) using logistic regression (indicated by the blue line). The shaded region indicates the 95% confidence interval of the predicted proportion. The trend was significantly positive for *Pseudomonas aeruginosa* (OR = 1.17; 95% CI, 1.08-1.31) and *Staphylococcus aureus* (OR = 1.08; 95% CI, 1.03-1.15), and it approached significance for *Enterococcus faecalis* (OR = 1.05; 95% CI, 0.98-1.16). The results demonstrate that an increased relative abundance of one of these species within a sample subsequently increases the likelihood that the species will be cultured.

Many of the bacteria that were identified by culture contributed to a large portion of the relative abundance of bacteria in the samples, but about half of the bacteria that were determined to comprise a dominant portion of the microbiota were not detected by culture (Figure 2). Some of the uncultured bacteria were obligate anaerobes (Figure 1 & Table 3), and the culture of obligate anaerobes was not attempted in this study because anaerobic cultures were not part of the patients’ standard of care. Obligate anaerobic bacteria were frequently identified by molecular testing, but they comprised an average of only 15.5% of the relative abundance in each sample. Previous studies have established that obligate anaerobes are important but difficult to detect in chronic wounds [2,13]. In this study, molecular testing identified an average of 84.5% (±27.5%) of the relative abundance of the bacteria as aerotolerant, but many of these bacteria were facultative anaerobes and may have been growing in anaerobic conditions.

In general, the likelihood of an organism being cultured was positively related to its relative abundance (Figure 3). This trend was evident when all aerotolerant genera were examined together (OR = 1.05, 95% CI 1.04-1.07). Culturing detected 54.9% of the relative abundance (as determined by 16S sequencing) of the potentially culturable bacteria, and the remaining 45.1% of the relative abundance went undetected by culturing.

Similar results were identified at the species level: species with a higher relative abundance in the sample as determined by molecular testing were more likely to be cultured (Figure 4). Three species were examined. The trend was significantly positive for *Pseudomonas aeruginosa* (OR = 1.17; 95% CI, 1.08-1.31) and *Staphylococcus aureus* (OR = 1.08; 95% CI, 1.03-1.15), and approached significance for *Enterococcus faecalis* (OR = 1.05; 95% CI, 0.98-1.16).

Exhaustively culturing all of the bacteria within a clinical sample has proven difficult [8,10,14]. Detecting the presence of anaerobes can be especially cumbersome, and anaerobes routinely are not identified, even though anaerobes are common contributors to the sample [8,13,14]. Additionally, many chronic infections include biofilms with dormant bacteria (viable but non-culturable [VBNC]), which are inherently difficult to culture [2,15]. Culturing from a mixed sample of bacteria, which is common in clinical wound samples, results in culture bias where some bacteria are positively selected because they grow quickly and robustly in the culture media, but fastidious organisms can be negatively selected and may grow more slowly or not at all. Therefore, using standard culture results to extrapolate the relative or absolute quantity of a bacterial species that was present within the original sample should not be performed because this method is unreliable (Figure 2).

Molecular methods can overcome some of cultures’ shortcomings. Molecular testing can detect biofilm bacteria, anaerobes, and VBNC bacteria regardless of how well the bacteria may or may not grow in culture [10,12]. Molecular methods can be designed to examine these organisms quantitatively, and, unlike culture testing, the presence of antibiotics within the sample does not interfere with the testing sensitivity. Molecular methods can elucidate the presence of bacteria regardless of whether or not the bacteria can be cultured.

Forty-six (46) culture isolates were cross-checked for identification using molecular methods, and discrepancies were observed in 20% (9/46) of the isolates (Table 2): 4 genus-level discrepancies and 5 species-level discrepancies. Of the 46 isolates, 43 were identified to species and 3 were only identified to genus. The 5 species-level discrepancies were arguably clinically insignificant. The four genus-level discrepancies were more clinically significant. Twice, biochemical testing determined isolates were staphylococci, but molecular testing determined the isolates were enterococci. Once, *P. aeruginosa* was identified by culture, but *Salmonella enterica* was identified by molecular testing. As mentioned previously, identification of bacteria by genotype is considered superior to the identification of bacteria by phenotype, so culture testing reported significant misidentifications of bacterial taxa 7% (3/46) of the time.

Limitations of this study include the small size of the sample population, the lack of anaerobic bacterial cultures, and the difficulty of replicating this study at other laboratories. Only 51 subjects were examined in this study, which was large enough to recognize statistically significant trends, but studies that analyze more samples should be performed. Anaerobic cultures were not obtained as this was a standard of care study. Many wound care specialists do not routinely request anaerobic cultures because the cost is significant, and organisms are rarely recovered. The molecular methods employed by in this study are available in very few clinical testing laboratories because of the high initial set-up costs and the large amount of data analysis that is required, which makes repeating this study at other institutions difficult.

The authors have performed previous studies to examine the clinical outcomes of patients when using molecular microbial diagnostic testing and have reported improvement in patient outcomes when using molecular testing when compared to when culture testing was used [16,17]. The current study may help to explain why this improvement was observed. Culture-free 16S DNA sequencing detects more of the bacterial components of the wound microbiota, and it is able to determine the relative abundance scores of the bacteria comprising the polymicrobial infection. The increased sensitivity and the relative abundance scores are helpful in guiding clinical decision making. It is appropriate to perform prospective studies that compare culture and molecular methods of bacterial identification and their impact on clinical decision making and patient outcome.

## Conclusion

This study suggests that bacteria with higher relative abundance scores (as determined by molecular testing) are more likely to be detected by culture, and bacteria with lower relative abundance scores are less likely to be detected by culture. Culture testing is not as sensitive as molecular testing, so culturing often fails to detect bacteria, even bacteria that are present in a high relative abundance. Bacteria that are present in high relative abundance are likely of high clinical relevance, including the bacteria that are not detected by culture. By considering the relative abundance scores when interpreting molecular results, clinicians can gain a clearer picture as to which bacteria may be most clinically relevant.

Prospective studies comparing patient and wound outcomes when using culture testing versus molecular microbial testing have not yet been performed, but retrospective studies suggest molecular microbial testing can improve patient outcomes [16,17].

## Competing interests

DDR has formerly been employed by WCC and RTL. SBC, EJR, and YS are employed by RTL. RDW is the Medical Director of WCC, and has equity positions in RTL and PGL. PGL & RTL engage in business transactions. PGL has applied for patents regarding molecular microbial testing.

## Authors’ contributions

EJR helped to acquire and analyze the data and draft the manuscript. DDR & SBC analyzed and interpreted the data and drafted the manuscript. YS performed molecular testing and participated in drafting the manuscript. RDW designed the study and participated in drafting the manuscript. All authors read and approved the final manuscript.

## Authors’ information

DDR is a Medical Laboratory Scientist and a Clinical Pathology resident. SBC is a Ph.D. biologist and statistician. YS is a Ph.D. molecular scientist. EJR is a Ph.D. computer scientist and bioinformatics researcher. RDW is a medical doctor and key opinion leader in the wound care community. He focuses his efforts on improving patient care by developing and using biofilm-based wound care practices.

## Pre-publication history

The pre-publication history for this paper can be accessed here:

http://www.biomedcentral.com/1471-2334/12/321/prepub

## Supplementary Material

Additional file 1 Bacteria identified by 16S and culture testing according to each subject. Click here for file
